# Gemini Surfactants: Advances in Applications and Prospects for the Future

**DOI:** 10.3390/molecules30234599

**Published:** 2025-11-29

**Authors:** Iwona Kowalczyk, Adrianna Szulc, Bogumił Brycki

**Affiliations:** Department of Bioactive Products, Faculty of Chemistry, Adam Mickiewicz University Poznan, 61-614 Poznan, Poland; iwkow@amu.edu.pl (I.K.); adrszu@gmail.com (A.S.)

**Keywords:** gemini surfactants, biofilm eradication, biocorrosion inhibitors, nanoparticles, oil industry

## Abstract

Cationic gemini surfactants, which constitute a unique class of amphiphilic molecules composed of two hydrophilic ammonium groups and two hydrocarbon tails connected by a spacer, have emerged as highly versatile functional agents with superior interfacial activity and self-assembly behavior compared to conventional monomeric analogs. Their structural tunability enables precise control over physicochemical properties, making them attractive for applications across diverse scientific and industrial domains. In biomedical sciences, gemini surfactants act as potent antimicrobial and anti-biofilm agents, as well as efficient carriers for drug and gene delivery. In nanotechnology and optoelectronics, they facilitate the synthesis and stabilization of nanoparticles, quantum dots, and perovskite nanocrystals, leading to improved colloidal stability, enhanced photophysical performance, and extended material lifetimes. Within the petroleum industry, gemini surfactants have proven effective in enhanced oil recovery (EOR) by reducing interfacial tension and in crude oil transportation as drag-reducing agents (DRAs), significantly lowering viscosity, turbulence, and pipeline energy losses. This review summarizes recent advances in the chemistry, mechanisms of action, and applications of gemini surfactants, highlighting their multifunctionality and emphasizing their potential in the development of next-generation sustainable technologies.

## 1. Introduction

Surfactants, also referred to as surface-active agents, represent a broad and versatile class of amphiphilic molecules characterized by the presence of both a hydrophilic (polar) head group and a hydrophobic (nonpolar) tail group. This dual structural motif endows surfactants with the ability to adsorb at interfaces and reduce surface and interfacial tension, thereby profoundly modifying the physical and chemical properties of multiphase systems. Beyond this fundamental property, surfactants exhibit diverse behaviors such as micellization, solubilization of poorly soluble substances, wetting, detergency, and stabilization of foams and emulsions. These unique attributes have made surfactants indispensable in a wide spectrum of applications, ranging from household and industrial cleaning formulations, cosmetics, and pharmaceuticals to more advanced uses in materials science, environmental engineering, petroleum recovery, and nanomedicine [1,2,3,4,5].

The origins of surfactant chemistry are closely tied to the discovery and use of soaps, produced from the saponification of natural fats and oils, which can be traced back to antiquity. The industrial revolution and subsequent advances in organic chemistry in the 19th and 20th centuries enabled the large-scale synthesis of synthetic surfactants, dramatically expanding the diversity and availability of these compounds. Over time, surfactants came to be classified according to the charge of their hydrophilic head groups: anionic surfactants (e.g., alkyl sulfates, sulfonates, carboxylates), which dominate the detergent industry; cationic surfactants (e.g., quaternary ammonium compounds), widely used for antimicrobial and conditioning applications; nonionic surfactants (e.g., alcohol ethoxylates, sorbitan esters), valued for their mildness and stability; and zwitterionic surfactants (e.g., betaines), which combine both positive and negative charges within the same molecule and display unique compatibility profiles. This classification underpins the rational design of surfactant systems tailored to specific technological and environmental demands [2,4].

In recent years, the development of surfactants has increasingly emphasized multifunctionality and sustainability [6]. While conventional surfactants have long been valued for their ability to lower interfacial tension, solubilize hydrophobic compounds, and stabilize dispersions, modern technological and environmental challenges require them to fulfill more complex roles. Emerging research highlights the potential of surfactants designed not only as interfacial agents but also as carriers for bioactive molecules, antimicrobial agents, corrosion inhibitors, or responsive materials capable of adapting to changes in pH, temperature, or ionic strength. At the same time, the environmental footprint of surfactants has become a critical concern, prompting a strong interest in biosurfactants produced by microorganisms or derived from renewable resources [7,8,9,10]. Such compounds typically combine effective surface activity with low toxicity, biodegradability, and a favorable ecological profile. The integration of multifunctional capabilities with eco-friendly characteristics is expected to shape the next generation of surfactant systems, ensuring both technological advancement and alignment with principles of green chemistry [6].

**Figure 1:**
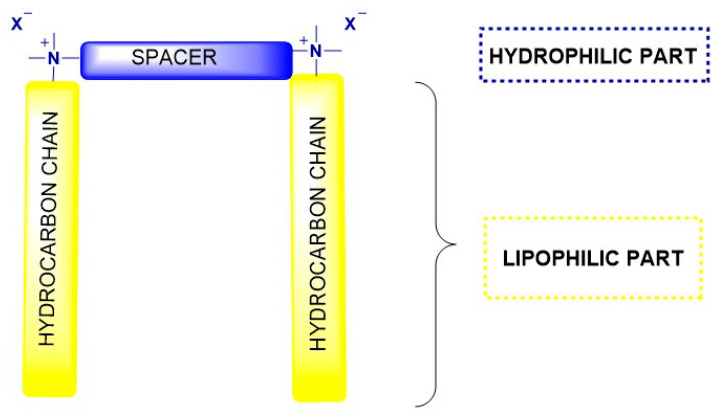
Schematic structure of gemini surfactants. The substituents and spacer may include chemical modifications depending on the desired property of the gemini surfactant.

Recently attention has increasingly been shifting towards novel surfactant architectures that surpass the performance and address the limitations of conventional monomeric surfactants. Among these, cationic gemini surfactants have emerged as one of the most promising innovations [11]. Structurally, gemini surfactants are composed of two hydrophilic head groups and two hydrophobic tails, covalently linked by a flexible or rigid spacer (Figure 1). This distinctive design results in much lower critical micelle concentrations (CMCs), enhanced surface activity, and a greater propensity to form diverse self-assembled structures compared with their conventional counterparts.

Moreover, the architecture of gemini surfactants is highly tunable: the length of the hydrophobic chains, the nature of the head groups, and the character of the spacer can be systematically modified to optimize properties for targeted applications [12,13,14,15]. These molecular advantages translate into remarkable performance in areas such as the solubilization of hydrophobic drugs, gene delivery, antimicrobial formulations, corrosion inhibition, and for use as building blocks for advanced nanomaterials. Importantly, the superior efficiency of gemini surfactants often allows for lower dosages, which can reduce environmental burden and improve sustainability—a critical consideration given the increasing emphasis on green chemistry and biodegradability in surfactant science [11,16,17,18].

Given these emerging perspectives, the study of gemini surfactants represents a rapidly growing frontier in colloid and interface science. Their structural versatility, multifunctionality, and potential to replace or complement traditional surfactants position them as key candidates for next-generation technologies. The present review aims to provide an overview of current knowledge on surfactants, to trace their historical development and classification, and to focus particularly on the unique features, applications, and future opportunities associated with gemini surfactants.

## 2. Results and Discussion

**Figure 2:**
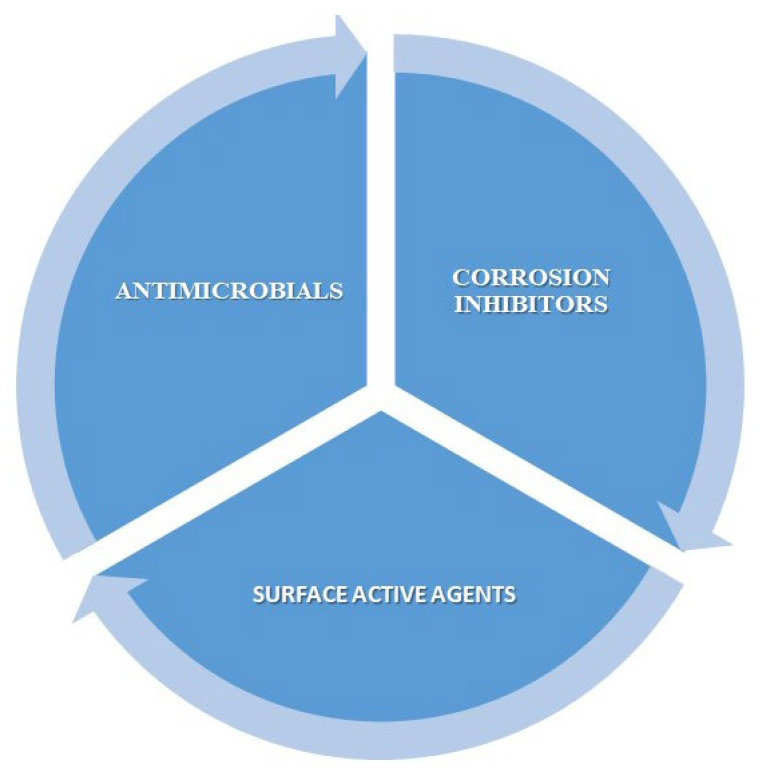
Schematic representation of the multifunctional properties of gemini surfactants.

Gemini surfactants find application in a wide spectrum of established and emerging technologies due to their exceptional surface activity and multifunctional properties (Figure 2).

In cleaning and formulation science, they are used as efficient emulsifiers, wetting agents, and solubilizers, enhancing detergency and stability in household and personal care products. Their role in environmental and industrial processes includes enhanced oil recovery, wastewater treatment, and corrosion inhibition. More recently, research has highlighted their potential in advanced applications such as biofilm elimination, where their strong membrane-disrupting activity can prevent microbial adhesion; in pipeline transport, where they reduce turbulence and drag in crude oil flow; in nanotechnology, as templates for nanoparticle synthesis with precise size and shape control; and in optoelectronics, where their ability to form ordered supramolecular structures supports the development of novel functional materials. In pharmaceuticals and biotechnology, they improve drug solubility and delivery, stabilize biomolecules, and act as antimicrobial agents. These modern directions underscore the versatility of gemini surfactants and their growing relevance across scientific and industrial fields [11,15,19].

### 2.1. Eradication of Biofilm by Gemini Surfactants

Biofilms are structured microbial communities embedded in a self-produced extracellular polymeric substance (EPS) matrix which adheres to living tissues and inert surfaces alike. They are formed by numerous bacterial species, including *Pseudomonas aeruginosa*, *Staphylococcus aureus*, *Escherichia coli*, and *Enterococcus faecalis*, as well as by fungi such as *Candida albicans*. The biofilm mode of growth provides microorganisms with enhanced resistance to antimicrobial agents, environmental stressors, and host immune responses, leading to persistent infections and contamination. Biofilm development is a significant problem in healthcare (catheters, prosthetic devices, ventilators, contact lenses), the food and pharmaceutical industries (pipelines, storage tanks, processing lines), and water distribution systems, where microbial colonization results in contamination, reduced efficiency, and material degradation [20,21,22,23,24].

Gemini surfactants have gained attention as potent anti-biofilm agents due to their unique amphiphilic structure and superior interfacial activity compared to conventional surfactants. Their mechanism of action involves several complementary processes. First, they disrupt the EPS matrix through interactions with polysaccharides and proteins, leading to increased porosity and reduced cohesion of the biofilm structure. Second, they integrate into microbial cell membranes, causing increased permeability and leakage of intracellular components, ultimately resulting in cell death [17]. Third, some gemini surfactants have been shown to interfere with quorum sensing pathways, thereby inhibiting microbial communication and preventing the maturation of biofilms [25]. Additionally, their adsorption to surfaces can hinder the initial adhesion of microbial cells, providing a preventive anti-biofouling effect [11].

Koziróg et al. investigated the activity of a gemini surfactant, hexamethylene-1,6-bis-(*N,N*-dimethyl-*N*-dodecylammonium bromide) 12-6-12, against *Asaia lannensis*, a spoilage bacterium in the beverage industry. The gemini surfactant showed significantly lower MIC (minimal inhibitory concentration) values than its monomeric analog (*N,N*-dimethyl-*N*-dodecylammonium bromide, DTAB) and effectively eradicated mature biofilms on polypropylene surfaces. At 10× MIC, 12-6-12 reduced biofilm viability by 91.8% after 4 h and achieved complete eradication after 24 h, highlighting its potential as a potent anti-biofilm agent in industrial settings [26]. In their subsequent research, Koziróg et al. provided direct comparative analysis between a 12-6-12 and its monomeric analog against an environmental strain of *Pseudomonas aeruginosa*. Gemini surfactant 12-6-12 displayed an MIC value 70 times lower than DTAB, highlighting its vastly superior potency. In biofilm eradication assays on polypropylene surfaces, 12-6-12 at 20MIC completely eradicated a mature biofilm within 4 h, a feat that took the monomeric surfactant 24 h. Furthermore, the study offered unique insights into the sub-MIC effects of these surfactants, showing that gemini compounds induced significant changes in the enzymatic and protein profiles of biofilm cells, disrupting key virulence factors and cellular processes, which monomeric surfactants failed to do to the same extent [27]. Mazurkiewicz et al. demonstrated that newly synthesized double quaternary ammonium salts with varying alkyl chain lengths (C12, C14, C16) and counterions (e.g., bromide, chloride, methylcarbonate, lactate, acetate) exhibit strong anti-biofilm activity against *C. albicans*. Notably, dibromides and dihydrochlorides with C12 chains showed the highest activity against planktonic cells, while C16-chain compounds were most effective in eradicating mature biofilms, achieving up to 80% destruction. These surfactants also significantly inhibited fungal adhesion to medical-relevant surfaces such as silicone, stainless steel, and glass [28]. Complementing these findings, Hsu et al. investigated a gemini quaternary ammonium compound with a functionalized spacer with the amine group, which exhibited exceptional broad-spectrum antifungal activity, including against drug-resistant *C. albicans*. The product with the dodecyl substituent not only inhibited planktonic growth but also significantly reduced biofilm formation and yeast-to-hyphal transition—a key virulence factor in *C. albicans*. Using transcriptomic and genetic screening approaches, the authors revealed that the compound’s mechanism of action involves the disruption of iron ion homeostasis, a pathway critical for biofilm integrity and fungal virulence. This novel mode of action highlights the potential of gemini surfactants to target essential physiological processes beyond membrane disruption [29].

In a more recent study, Hafidi et al. synthesized gemini arginine-based surfactants and evaluated their anti-biofilm activity against MRSA (Methicillin-Resistant *Staphylococcus Aureus*) and *Candida* species. These compounds, particularly those with C_5_–C_6_ alkyl chains, demonstrated strong biofilm eradication at low concentrations (32–64 µg/mL). The study also noted that the formation of large aggregates (e.g., vesicles) by more hydrophobic analog (C_8_–C_10_) may reduce their anti-biofilm efficacy, underscoring the importance of optimizing alkyl chain length for maximum activity [30]. Labena et al. synthesized a cationic gemini surfactant with pyridine rings and evaluated its multifunctional properties, including broad-spectrum antimicrobial, anti-biofilm, and biocidal activities, particularly in high-salinity environments relevant to the petroleum industry. The surfactant also demonstrated significant anti-biofilm activity, inhibiting biofilm formation and eradicating pre-formed biofilms of both aerobic and anaerobic sulfidogenic bacteria at concentrations of 0.31–0.62 mM [31].

Together, these studies indicate that gemini surfactants are highly effective in biofilm prevention and eradication, with their activity being influenced by alkyl chain length, counterion type, and molecular flexibility. Their low cytotoxicity and high biocompatibility further support their potential applications in medical, industrial, and environmental settings. These properties make gemini surfactants promising alternatives or adjuncts to conventional disinfectants and antibiotics, particularly in scenarios where biofilms pose persistent challenges (Table 1). Current research continues to explore the structural optimization of gemini surfactants to maximize antimicrobial potency while minimizing toxicity (reducing the use concentration of the biocide) in comparison to monomeric quaternary ammonium salt, underscoring their growing role in modern biofilm management strategies [32,33].

Gemini surfactants have also demonstrated notable antiviral activity against both influenza viruses and SARS-CoV-2. Their dual-cationic structure enables strong electrostatic and hydrophobic interactions with viral envelopes, leading to disruption of the lipid bilayer and denaturation of surface glycoproteins. These findings highlight their potential as effective antiviral agents for disinfection and surface protection against enveloped viruses [34,35].

### 2.2. Gemini Surfactants as Biocorrosion Inhibitors

**Figure 3:**
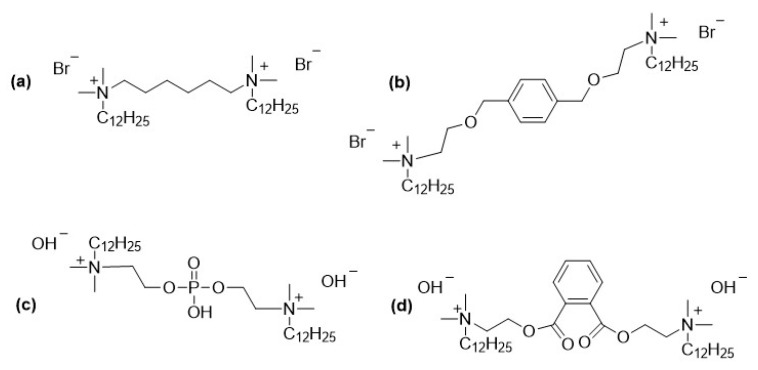
Gemini surfactants described as biocorrosion inhibitors: (**a**) [43]; (**b**) [44]; (**c**) [45]; (**d**) [46].

Microbiologically influenced corrosion, commonly referred to as biocorrosion, is a degradation process of metals and alloys caused by the metabolic activity of microorganisms. Bacteria, archaea, and fungi are the main groups involved, with sulfate-reducing bacteria (e.g., *Desulfovibrio vulgaris*, *Desulfotomaculum nigrificans*), iron-oxidizing bacteria (e.g., *Gallionella ferruginea*, *Leptothrix ochracea*), and acid-producing microorganisms (e.g., *Acidithiobacillus ferrooxidans*, *Acidithiobacillus thiooxidans*) playing particularly significant roles. Other species, such as *Pseudomonas aeruginosa* and *Shewanella putrefaciens*, are also known to contribute to biofilm formation and metal deterioration. Biocorrosion affects a wide range of industrial sectors, including oil and gas, water treatment, shipping, and power generation, leading to severe material losses, infrastructure damage, and high economic costs worldwide. The annual expenses associated with biocorrosion are estimated at billions of dollars due to equipment replacement, process downtime, and environmental hazards. Protective strategies include material selection, surface modification, cathodic protection and importantly, the use of biocorrosion inhibitors [36]. Among the most promising chemical approaches are gemini surfactants (Figure 3), which exhibit enhanced surface activity and antimicrobial properties, making them effective candidates for mitigating biocorrosion [37,38,39,40,41,42].

Pakiet et al. investigated a cationic gemini surfactant (12-6-12), as a dual-function agent against corrosion and biocorrosion on pre-phosphated mild steel. In a standard 3.5% NaCl solution, the gemini surfactant showed exceptional corrosion inhibition efficiency (>95%) even at very low concentrations (0.01 mM), with efficiency reaching 99.3% at 2 mM. The inhibition mechanism followed the Langmuir adsorption isotherm, indicating the formation of a protective monolayer on the steel surface. Its biocidal efficacy was tested against the sulfate-reducing bacterium (SRB) *Desulfovibrio salexigens* in anaerobic Postgate’s B medium. The surfactant exhibited a very low MIC of 0.018 mM, completely preventing SRB growth. In biocorrosion experiments, the surfactant not only inhibited bacterial cultivation but also effectively suppressed the corrosion process induced by SRB, maintaining high polarization resistance throughout the 12-day test. The study concludes that the12-6-12 is a highly efficient multifunctional compound, acting as a potent corrosion inhibitor and a powerful biocide against SRB at remarkably low concentrations, making it a promising candidate for protecting industrial infrastructure from microbiologically influenced corrosion [43].

Based on the study by Zhu et al., the gemini surfactant 12-B-12, containing a semi-rigid spacer with a benzene ring and ether oxygen atoms, demonstrated exceptional performance as both a corrosion inhibitor and a biocide against SRB in simulated seawater. The compound exhibited a remarkably low CMC of 0.01 mM and achieved an inhibition efficiency of up to 98.3% after 30 days of exposure, effectively reducing both planktonic and sessile SRB to undetectable levels. Its dual functionality stems from strong adsorption on carbon steel surfaces via multiple active sites (quaternary ammonium groups, π-electrons, and lone pairs on oxygen atoms), forming a protective hydrophobic layer, while also disrupting bacterial membranes. These properties make 12-B-12 a promising candidate for multifunctional coatings or additives in marine environments to mitigate biocorrosion in infrastructures such as pipelines, offshore platforms, and ship hulls [44].

Labena et al. synthesized and characterize a cationic gemini surfactant with a phosphoryl group in the spacer and hydroxides as counterions. The study evaluates its dual function as a corrosion inhibitor and biocide against SRB (*Desulfovibrio* spp.) isolated from a high-salinity (5.49% NaCl) oil-field water tank. The compound demonstrated significant surface activity with a low CMC (2.3 mM). At 5 mM concentration, it achieved 97% corrosion inhibition efficiency and completely suppressed sulfide production and biofilm formation. The surfactant exhibited nonspecific biocidal activity against both Gram-Positive (*S. aureus*) and Gram-Negative (*P. aeruginosa*, *E. coli*) bacteria. The mechanism involves electrostatic interaction with bacterial membranes and physical disruption leading to cell death, as well as adsorption onto metal surfaces forming a protective layer. The study concludes that this gemini surfactant is a promising candidate for controlling biocorrosion in high-salinity industrial environments due to its strong inhibitory and biocidal properties [45]. In another papers the same authors characterized cationic gemini surfactants with a phthaloyl group (ester and benzene ring) in the spacer. This gemini surfactant exhibited superior surface-active properties (indicating a high tendency for adsorption at interfaces) Its biocidal activity was tested against a complex environmental sulfidogenic microbial community (enriched from an Egyptian petroleum company’s water tank) comprising SRB (e.g., *Desulfovibrio* and *Desulfotomaculum genera*) and methanogenic archaea. The study demonstrated that the gemini surfactant effectively inhibited both planktonic cells and biofilms. At a concentration of 1 mM, it completely inhibited sulfide production (a key corrosion metabolite) and bacterial growth (MIC = 1 mM), achieving a metal corrosion inhibition efficiency of 95% on mild steel. The study concludes that this gemini surfactant is a highly effective dual-function agent, acting as both a powerful corrosion inhibitor by forming a protective adsorbed film on the metal surface and a broad-spectrum biocide against problematic sulfidogenic bacteria in industrial settings, such as petroleum production [46,47].

Biocorrosion represents a significant challenge for industrial infrastructure, as microorganisms such as sulfate-reducing bacteria, iron-oxidizing bacteria, and acid-producing species accelerate metal degradation through biofilm formation and metabolite production. Recent studies demonstrate that gemini surfactants are highly promising multifunctional agents capable of both inhibiting electrochemical corrosion and suppressing microbial activity, particularly of *Desulfovibrio* spp. and related sulfidogenic communities. Various structural modifications, including semi-rigid spacers, triazine moieties, and phosphoryl or phthaloyl groups, have been shown to enhance adsorption efficiency, reduce CMC, and improve broad-spectrum antimicrobial activity. The dual mechanism of surface protection and microbial inhibition allows gemini surfactants to achieve outstanding performance at remarkably low concentrations, even under harsh conditions such as high salinity or simulated seawater. These findings highlight their potential as next-generation inhibitors for mitigating biocorrosion in critical sectors such as oil and gas, water treatment, and marine environment.

### 2.3. Biomedical Applications of Gemini Surfactants

**Figure 4:**
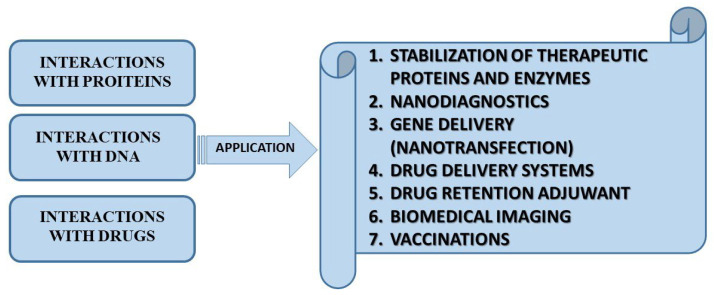
Potential bioapplications for gemini surfactants.

Gemini surfactants have emerged as highly versatile molecules in medical research and pharmaceutical technology due to their unique structural and physicochemical properties. Their exceptionally low CMC, strong surface activity, and notable ability to self-assemble enable efficient interactions with biological membranes or with DNA. Moreover, their cationic nature and tunable molecular design provide enhanced compatibility with therapeutic agents. These features make gemini surfactants attractive candidates for diverse medical applications in drug and gene delivery systems, transfection agents, and protective coatings for biomedical devices. Their multifunctionality, combining both biocidal activity and the capacity to act as nanocarriers, positions gemini surfactants as promising tools for advancing modern therapies and improving the performance of biomedical materials (Figure 4) [15,48]. Based on Shalan et al., gemini surfactants show promising potential in oncology due to their direct cytotoxic effects on cancer cells. In this study, three newly synthesized cationic gemini surfactants with different alkyl chain lengths (C4, C6, C8) were tested against the human breast cancer cell line MCF-7. The compounds displayed strong surface activity and low critical micelle concentration, with cytotoxicity depending on chain length. These findings suggest that gemini surfactants can be developed as selective anticancer agents, offering a novel therapeutic strategy for breast cancer treatment [49].

Gene transfer is essential because many diseases originate from defective or dysregulated genes. By introducing functional DNA or RNA into target cells, gene transfer can replace or repair faulty genes, silence harmful ones, or introduce new genetic functions. This enables the long-term treatment of genetic disorders, cancers, and infectious diseases at their root cause, rather than only the alleviation of symptoms. Gemini surfactants have emerged as promising non-viral gene delivery vectors due to their unique structural properties. Typical gemini surfactants like α,ω-polymethylene-bis(*N*-dodecyl-*N,N*-dimethylammonium bromides) [50] or imidazolium- and pyridinium-based ones [51], effectively condense nucleic acids (e.g., plasmid DNA, siRNA, mRNA) via electrostatic interactions and hydrophobic forces, forming stable nanocomplexes. The mechanism involves initial electrostatic binding between gemini surfactants monomers/micelles and negatively charged DNA, followed by hydrophobic interactions that enhance complex stability. These complexes facilitate cellular uptake through endocytic pathways (e.g., clathrin- or caveolae-mediated endocytosis), with some formulations enabling endosomal escape via proton sponge effects or membrane fusion. These systems have demonstrated potential applications in treating cancers (e.g., melanoma, glioblastoma), liver diseases (e.g., HBV therapy using CRISPR/Cas9), heart disorders (e.g., myocardial hypertrophy), glaucoma (e.g., gene delivery to retinal cells), and mucosal or enamel defects. Optimization strategies—such as spacer length adjustment, hydrophobic tail elongation, head group modification (e.g., aromatic heterocycles), and functionalization with peptides, amino acids, or biomimetic coatings—enhance transfection efficiency and biocompatibility, making gemini surfactants versatile candidates for targeted gene therapy [52,53,54,55].

The ability of gemini surfactants to interact effectively with proteins is of high significance, as it directly influences protein stability, conformational integrity, and functional activity. These interactions can modulate protein folding and aggregation, protect biomolecules from enzymatic degradation, and facilitate their incorporation into delivery systems. Consequently, gemini surfactants provide a versatile platform for enhancing the bioavailability and therapeutic efficacy of protein-based drugs [56,57,58,59]. Gemini surfactants have strong potential in the treatment of neurodegenerative diseases such as Alzheimer’s and Parkinson’s due to their ability to form stable, nanoscale complexes with therapeutic nucleic acids or proteins and facilitate their transport across biological barriers. Their tunable cationic structure enables the efficient condensation and protection of neuroprotective peptides, while also promoting endosomal escape and cellular uptake in neuronal cells. Importantly, gemini-based nanocarriers could be engineered to cross the blood–brain barrier, a major obstacle in neuropharmacology, thereby enabling the targeted delivery of gene therapies or disease-modifying biomolecules directly to the central nervous system. Such approaches may support strategies aimed at silencing pathogenic proteins (e.g., α-synuclein in Parkinson’s or β-amyloid in Alzheimer’s) or restoring neuroprotective pathways, offering a promising route toward disease-modifying treatments [60]. Moreover, gemini surfactants can enhance vaccines by stabilizing and efficiently delivering nucleic acids or antigens, improving immune response while reducing toxicity compared to viral carriers. In biosensors, their strong self-assembly and ability to interact with biomolecules enable the construction of sensitive nanostructured interfaces, increasing detection accuracy for diagnostic applications [61,62].

Gemini surfactants are highly attractive for applications in drug delivery, where they function both as primary drug carriers and as membrane adjuvants in liposomal formulations [61]. Cationic gemini surfactants can form self-assembled catanionic vesicles directly with anionic hydrotropic drugs, without the need for phospholipids. This is a powerful method to create drug-loaded nanovehicles. The anionic drug (e.g., diclofenac sodium) electrostatically binds to the cationic headgroups of the geminis. This charge neutralization reduces headgroup repulsion, favoring tighter packing and a transition from spherical micelles to larger bilayered structures [63]. In conclusion, gemini surfactants are highly versatile tools in nanomedicine. Their ability to act as both the primary building blocks of drug carriers and as functional adjuvants to enhance the performance of liposomes makes them a promising platform for the delivery of a wide range of therapeutic agents, including statins (atorvastatin), non-steroidal anti-inflammatory drugs (diclofenac), and chemotherapeutics (doxorubicin) [64].

### 2.4. Gemini Surfactants in Nanotechnology

**Figure 5:**
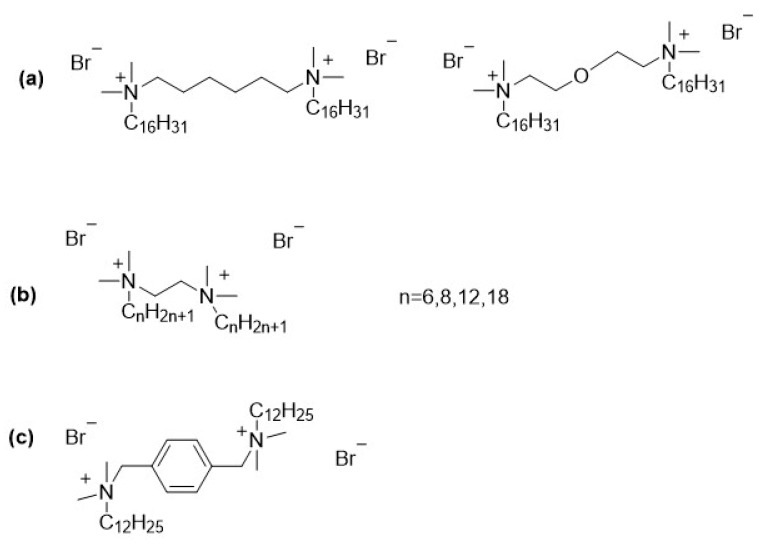
Examples of gemini surfactants described as nanoparticle stabilizers: (**a**) [65]; (**b**) [66]; (**c**) [67].

Gemini surfactants have found wide-ranging applications in the controlled synthesis of nanoparticles and nanomaterials (Figure 5), where they function as both stabilizing and structure-directing agents. In the case of noble metals such as gold, silver, and palladium, gemini surfactants are used during wet-chemical reduction to guide the nucleation process and to limit uncontrolled growth, resulting in nanoparticles with narrower size distributions and well-defined morphologies [65,66,67,68,69]. For semiconductor quantum dots like CdSe, ZnS, and PbS, gemini surfactants not only prevent agglomeration in colloidal suspensions but also passivate surface trap states, which directly enhances the photoluminescence efficiency and stability of the nanocrystals [70,71]. A particularly promising area is the use of gemini surfactants in the synthesis of lead halide perovskite nanocrystals (e.g., CsPbBr_3_), where they improve water resistance, extend operational lifetimes under illumination, and preserve the high quantum yield that is otherwise quickly lost due to surface degradation [72]. Beyond optical properties, gemini surfactants facilitate the dispersion of nanoparticles in polar and nonpolar solvents, making it possible to process nanomaterials into thin films or composite structures suitable for optoelectronic devices. Their presence has also been linked to better charge transport in nanostructured films, which is critical for applications in solar cells, light-emitting diodes, and photodetectors. Furthermore, in catalytic applications, gemini-stabilized metal nanoparticles exhibit higher activity and selectivity, attributed to their controlled surface exposure and resistance to aggregation under reaction conditions. Overall, the use of gemini surfactants in nanomaterial synthesis offers synergistic benefits: including: improved control over morphology, enhanced colloidal and chemical stability, and superior functional properties, making them an increasingly valuable component in the design of advanced materials for optoelectronics, energy conversion, sensing, and biomedicine [73].

Lead halide perovskite nanocrystals, such as CsPbBr_3_, are promising materials for optoelectronic applications, but their stability in the presence of moisture and light remains a significant challenge. Studies have shown that the use of cationic gemini surfactants, such as alkanediyl-α,ω-bis(dodecyldimethylammonium) bromide (12-n-12), significantly improves the stability of these nanocrystals. CsPbBr_3_ nanocrystals passivated with gemini surfactants exhibit enhanced resistance to water-induced degradation compared to those stabilized with conventional ligands like dimethyldodecylammonium oleate or oleic acid/oleyl amine [72]. In organic light-emitting diodes, the quality of interfaces between emissive layers and charge transport layers is critical for device performance. Gemini surfactants can function as interfacial modifiers, improving layer morphology, surface coverage, and charge injection, while reducing exciton quenching. This leads to enhanced efficiency and stability of OLED (Organic Light Emitting Diode) devices [74]. The use of gemini surfactants in the synthesis of nanomaterials for optoelectronics provides multiple advantages:

- Improved stability: Increased resistance to moisture, light, and other degrading factors.
- Morphology control: Precise control over nanoparticle size and shape.
- Enhanced optical performance: Higher fluorescence efficiency and reduced energy losses.
- Better dispersion: Facilitates fabrication of thin films and integration with other materials.

Overall, gemini surfactants play a key role in the design and production of advanced optoelectronic devices, including solar cells, LEDs, photodetectors, and bioimaging systems, by improving material stability, optical properties, and device performance [72,74,75,76].

### 2.5. Gemini Surfactants in Oil Recovery and Transportation

Gemini surfactants have attracted significant attention in the petroleum industry due to their unique ability to reduce interfacial tension, enhance oil displacement, and improve fluid flow in reservoirs and pipelines. Their dual-head and dual-tail structure, combined with low critical micelle concentrations, allows them to form highly efficient micellar and interfacial films, which promote the mobilization of trapped oil in porous media. Gemini surfactants are increasingly employed as surfactant flooding agents in enhanced oil recovery EOR) due to their superior surface activity and ability to form stable micelles at low concentrations. In surfactant flooding, an aqueous solution of the surfactant is injected into the reservoir to reduce the oil–water interfacial tension, thereby mobilizing trapped residual oil. Gemini surfactants, because of their unique dimeric structure, exhibit much lower critical micelle concentrations and stronger adsorption at the oil–water interface. This results in a significant reduction in residual oil saturation and improved sweep efficiency. Moreover, their molecular architecture provides enhanced tolerance to salinity, temperature, and hardness of the reservoir brine, making them suitable for harsh reservoir conditions. Studies have shown that their use can lead to higher oil recovery rates compared to conventional single-chain surfactants, particularly in reservoirs with high salinity and temperature conditions [18,77,78,79]. In addition to EOR, gemini surfactants play a critical role in crude oil transportation by stabilizing water-in-oil emulsions and preventing pipeline fouling. They reduce viscosity and modify the rheological behavior of heavy crude oils, enabling easier pumping and minimizing pressure drops. Moreover, their superior adsorption and interfacial properties enhance the stability of foams and microemulsions used in well stimulation, sand control, and drilling fluids. The tunability of gemini surfactant structures—available through variations in chain length, spacer type, and counterions—allows for the design of formulations optimized for specific reservoir conditions and crude compositions [18,80,81].

Table 2 compares the interfacial and recovery efficiencies of cationic gemini surfactants with those of conventional anionic, monomeric cationic, and zwitterionic/nonionic surfactants commonly used in enhanced oil recovery (EOR). The data clearly demonstrate that cationic gemini surfactants achieve substantially lower critical micelle concentrations and stronger interfacial tension (IFT) reduction—often below 1 mN/m and occasionally reaching the ultra-low range (10^−3^ mN/m)—at considerably lower concentrations (0.05–0.25 wt%). These physicochemical advantages translate into higher incremental oil recoveries, typically 11–27% of the original oil in place (OOIP) in laboratory core-flood experiments. By contrast, conventional anionic and monomeric cationic surfactants require higher dosages (0.1–1.0 wt%) to achieve comparable or slightly lower efficiencies, while zwitterionic and nonionic systems provide intermediate performance with superior salt and thermal stability. Despite their superior surface activity, cationic gemini surfactants may suffer from strong adsorption losses in clay-rich sandstones, suggesting that they are most effective in carbonate or high-salinity/high-temperature reservoirs, or when formulated in mixed systems that mitigate adsorption [80,82,83,84,85].

Gemini surfactants have garnered significant attention as effective drag-reducing agents (DRAs) in the pipeline transportation of crude oil, particularly in the context of heavy and waxy crude oils. Studies have demonstrated that the incorporation of gemini surfactants can lead to substantial reductions in crude oil viscosity and drag forces within pipelines. For instance, research indicates that the addition of gemini surfactants to crude oil can reduce viscosity by up to 85–92% at temperatures as low as 10 °C, facilitating smoother flow and reducing the energy required for pumping [86]. The mechanism by which gemini surfactants exert their drag-reducing effects involves the formation of viscoelastic wormlike micelles that interact with the turbulent eddies in the fluid flow. These micelles absorb and dissipate turbulent energy, leading to a transition from a turbulent to a more laminar flow regime. This transition is particularly beneficial for transporting heavy crude oils, which are prone to high viscosity and deposition issues in pipelines. Moreover, the structural design of gemini surfactants allows for the modulation of their drag-reducing properties through variations in the spacer group between the hydrophobic tails. For example, the introduction of bonds with heteroatom in the spacer region can enhance the stability and responsiveness of the surfactant, enabling controlled release and reorganization of the micellar structures under specific environmental conditions, such as changes in pH [83].

In summary, gemini surfactants offer a promising approach to enhancing the transportation of crude oils through pipelines. Their ability to reduce viscosity, suppress turbulence, and prevent deposition makes them valuable additives in the petroleum industry, particularly for challenging crude oil types. Ongoing research into the optimization of their chemical structure and formulation will likely lead to more efficient and sustainable solutions for crude oil transportation in the future.

## 3. Conclusions

Gemini surfactants represent a rapidly evolving class of amphiphiles with remarkable multifunctionality, capable of addressing diverse challenges across biomedical, environmental, materials science, and petroleum engineering domains. Their structural features—dual hydrophobic tails, dual cationic or anionic head groups, and variable spacers—confer exceptionally low critical micelle concentrations, enhanced interfacial activity, and superior capacity for self-assembly, which translate into significant performance improvements compared with conventional surfactants.

In biomedical sciences, gemini surfactants have demonstrated potent antibacterial and antibiofilm activities, disrupting microbial membranes and biofilm matrices more effectively than single-chain surfactants. Their strong antimicrobial action, combined with their tunable toxicity and biocompatibility, positions them as promising candidates for pharmaceutical formulations, wound treatment, and hygienic applications. Furthermore, their ability to condense nucleic acids and encapsulate hydrophobic drugs highlights their value in drug delivery and gene therapy, where they act as efficient non-viral vectors.

In the field of biocorrosion, gemini surfactants exhibit dual functionality as both surface protectants and biocides. By adsorbing strongly onto metallic surfaces, they form protective films that reduce microbial adhesion and electrochemical degradation, while their inherent antimicrobial activity suppresses the growth of sulfate-reducing and acid-producing bacteria. These combined mechanisms enable them to significantly mitigate microbiologically influenced corrosion, particularly in harsh saline and anaerobic environments relevant to oilfield pipelines, marine installations, and water treatment systems.

In nanotechnology and optoelectronics, gemini surfactants serve as both stabilizers and structure-directing agents in the synthesis of nanoparticles, quantum dots, and perovskite nanocrystals. Their use has been shown to improve colloidal stability, control particle size and morphology, and passivate surface trap states, resulting in enhanced photoluminescence quantum yield and prolonged device lifetimes. Applications in light-emitting diodes, solar cells, and photodetectors underscore their critical role in the advancement of high-performance optoelectronic devices.

In the petroleum industry, gemini surfactants provide multiple benefits. As enhanced oil recovery agents, they significantly reduce oil–water interfacial tension and alter wettability, facilitating the mobilization of trapped hydrocarbons even under high salinity and high-temperature conditions. As drag-reducing agents, they form viscoelastic micellar structures that suppress turbulence and lower frictional losses in pipelines, thereby improving flow efficiency and reducing energy consumption. In addition, their capacity to reduce viscosity and prevent wax deposition further enhances crude oil transport.

In our opinion despite their remarkable efficiency and versatility, the practical utilization of gemini surfactants is still constrained by several significant challenges. One of the primary issues lies in their multistep and often low-yield synthetic pathways, which involve complex purification procedures and the use of expensive or environmentally sensitive reagents. This not only increases production costs but also limits scalability and industrial feasibility. Moreover, the relationship between molecular architecture and functional properties remains insufficiently understood, necessitating extensive computational modeling and experimental optimization. Environmental and toxicological aspects also present unresolved issues, as data on their biodegradability, bioaccumulation potential, and long-term ecological impact are still limited. Consequently, while gemini surfactants hold great promise for applications in detergency, pharmaceuticals, and nanotechnology, overcoming these scientific and technological barriers remains essential for their widespread adoption.

Taken together, these advances illustrate the broad spectrum of gemini surfactant applications and their potential to deliver both technological and economic benefits. Beyond functionality, their structural versatility allows for tailored designs to meet the requirements of specific environments, from biological systems to industrial reservoirs. Future research should focus on optimizing molecular designs for targeted applications, evaluating biodegradability and environmental safety, and scaling up cost-effective production. With continued development, gemini surfactants are poised to play a central role in next-generation solutions for healthcare, energy, materials science, and sustainable industrial processes.

## Figures and Tables

**Table 1:** Summary of the possibility of the use of gemini surfactants in biofilm eradication in relation to strains and applications.

Biofilm-Forming Strains	Application	References
*A. lannensis*	food industry (beverage production)	[26]
*P. aeruginosa*	water installations	[27]
*C. albicans*	medical devices	[28,29,30]
Methicillin-Resistant *Staphylococcus aureus*	medical devices	[30]
*B. subtilis* and *E. coli*	petroleum industry	[31]

**Table 2:** Comparison of Surfactant Efficiency in EOR.

Surfactant Type	Typical Concentration Range	Typical IFT Achieved (Oil/Water)	Reported Incremental Oil Recovery [% OOIP]
cationic gemini	~500–2500 ppm (≈0.05–0.25 wt%)	Very strong interfacial activity; often reduces IFT to <1 mN/m, and in optimized blends even ultra-low IFT (10^−3^ mN/m)	~11–27 in lab/core-flood tests
anionic	~200–2000 ppm (0.02–0.2 wt%)	Good IFT reduction; ~0.6–1 mN/m	~5–20 in lab studies
cationic	~0.1–1.0 wt% (≈1000–10,000 ppm)	Strong IFT reduction; down to 0.33 mN/m	~14% in lab studies
zwitterionic/nonionic	~100–2000 ppm	Moderate–strong performance; IFT often <1 mN/m	~10–30 in lab studies

## Data Availability

The data presented in this study are available in the article.
